# “What We Know and What We Do Not Know about Evolutionary Genetic Adaptation to High Altitude Hypoxia in Andean Aymaras”

**DOI:** 10.3390/genes14030640

**Published:** 2023-03-03

**Authors:** Ricardo Amaru, Jihyun Song, N. Scott Reading, Victor R. Gordeuk, Josef T. Prchal

**Affiliations:** 1 Cell Biology Unit, School of Medicine, San Andres University, La Paz 0201, Bolivia; 2 Division of Hematology, School of Medicine, University of Utah, Salt Lake City, UT 84132, USA; 3 Department of Pathology-ARUP Laboratories, University of Utah, Salt Lake City, UT 84132, USA; 4 Department of Medicine, University of Illinois at Chicago, Chicago, IL 61820, USA

**Keywords:** high altitude, adaption, Aymara, erythrocytosis, SNPs

## Abstract

Three well-studied populations living at high altitudes are Tibetans, Andeans (Aymaras and Quechuas), and Ethiopians. Unlike Tibetans and Ethiopians who have similar hemoglobin (Hb) levels as individuals living at sea level, Aymara Hb levels increase when living at higher altitudes. Our previous whole genome study of Aymara people revealed several selected genes that are involved in cardiovascular functions, but their relationship with Hb levels was not elucidated. Here, we studied the frequencies of known evolutionary-selected variants in Tibetan and Aymara populations and their correlation with high Hb levels in Aymara. We genotyped 177 Aymaras at three different altitudes: 400 m (Santa Cruz), 4000 m (La Paz), and 5000 m (Chorolque), and correlated the results with the elevation of residence. Some of the Tibetan-selected variants also exist in Aymaras, but at a lower prevalence. Two of 10 Tibetan selected variants of *EPAS1* were found (rs13005507 and rs142764723) and these variants did not correlate with Hb levels. Allele frequencies of 5 Aymara selected SNPs (heterozygous and homozygous) at 4000 m (rs11578671_*BRINP3*, rs34913965_*NOS2*, rs12448902_*SH2B1*, rs10744822_*TBX5*, and rs487105_*PYGM*) were higher compared to Europeans. The allelic frequencies of rs11578671_*BRINP3*, rs34913965_*NOS2*, and rs10744822_*SH2B1* were significantly higher for Aymaras living at 5000 m than those at 400 m elevation. Variant rs11578671, close to the *BRINP3* coding region, correlated with Hb levels in females. Variant rs34913965 (*NOS2*) correlated with leukocyte counts. Variants rs12448902 (*SH2B1*) and rs34913965 (*NOS2*) associated with higher platelet levels. The correlation of these SNPs with blood cell counts demonstrates that the selected genetic variants in Aymara influence hematopoiesis and cardiovascular effects.

## 1. Introduction

Humans migrating out of Africa encountered new conditions, including living at high altitudes. Tibetans and Andean Aymaras have inhabited regions at elevations of 4000 m or more for approximately 44,000 and 14,000 years, respectively [1,2]. Evolutionary adaptations to high altitude in these geographically separated populations differ in Hb concentrations [3,4,5]. Tibetans living at 4000 m have similar hemoglobin (Hb) levels to individuals living at sea level, whereas Hb levels of Bolivian Andean Aymaras living at 4000 m are increased [6,7,8] and this difference is the subject of our report.

South America is home to more than 430 million people, composed of many ethnicities that have settled across widely different altitudes. In Bolivia, the Aymaras, with an estimated 2.3 million people, are one of two representative high-altitude populations in the Andes mountains. The Aymaras are an indigenous people, believed to have anciently occupied the high plateaus (Altiplano) of the central Andes in present-day western Bolivia, southern Peru, northern Chile and Argentina. Although a majority of Aymaras continue to live in this region, they have been migrating to live in regions ascending from 400 to 5000 m. Today, Aymaras can be found living at various, diverse elevations. For example, Santa Cruz at 400 m, La Paz at 4000 m, and Chorolque, a mining town at 5000 m. La Paz is in the Altiplano and is the highest capital city in the world. Its population resides across a broad range of high altitudes, from downtown, located at the bottom of the valley, at 3650 m, to the La Paz altiplano at about 4150 m. The Aymaras are thus uniquely situated to have developed evolutionary characteristics and provide an outstanding resource for the study of high-altitude adaptation.

The second-most populous high-altitude ethnic group of the Andes are the Quechuas, whose ancestors are attributed to having established the Inca Empire. The population of Quechua is estimated at 13 million people, who account for many of the indigenous ethnic groups in Perú, Ecuador, Argentina, and Chile; the Quechuas are the second largest ethnic group in Bolivia. These two well-defined high-altitude populations, the Aymaras and Quechuas, are distinct and immediately recognizable, having different habitus, languages and folklore. There is a common perception among local Andean populations that Quechuas live in the high Andes Mountain valleys, while Aymaras live on the top of the Andes mountains. While Aymaras and Quechuas are considered to have common ancestry, they must have been separated many generations ago. However, rigorous studies concerning their evolutionary separations do not exist. There are other, much smaller, ethnic groups residing in the Andes mountains as well [9].

Life in a high-altitude environment is challenging, with intense cold, higher UV exposure, extremely low humidity, and limited food resources affecting health and quality of life. In addition, barometric pressure decreases non-linearly with increasing altitude, resulting in a decreased partial pressure of oxygen in the air and fewer oxygen molecules being inhaled per breath. In Santa Cruz, the average barometric pressure is 719 mmHg and the atmospheric concentration of oxygen is 19.8%; in La Paz, the average barometric pressure is 453 mmHg and the oxygen concentration is approximately 12.7%; and in Chorolque, the average barometric pressure and oxygen concentration are 405 mmHg and 11.1%, respectively. Due to hypobaric hypoxia with increasing altitude, the human body undergoes numerous physiological and potentially pathological responses. Lowland residents that ascend to high altitudes acclimatize through increased ventilation, arterial oxygen partial pressure, and oxygen saturation, along with decreased total body water (resulting in reduced plasma volume and increased concentration of Hb) are key physiological components of high-altitude acclimatization [10,11,12,13]. Failure to adapt results in several high-altitude sicknesses: acute mountain sickness, high-altitude cerebral edema, and high-altitude pulmonary edema [14,15,16]. However, long-term exposure to high-altitude living can also induce a condition of chronic mountain sickness with symptoms of fatigue, shortness of breath, aches and pains, and cyanosis evident in the lips and skin. In affected individuals, the body attempts to overcompensate for hypoxia by excessive erythrocytosis frequently associated with pulmonary hypertension [6,8].

In comparison, the Tibetan Plateau has an average elevation of 4500 m (3000–5500 m) and has reportedly been occupied since neolithic times. These indigenous people have been exposed to this high-altitude, hypoxic environment for more generations and have resided at higher altitudes than the Andean high mountain inhabitants: Aymara and Quechuas [1,17]. The combination of low oxygen pressure at high altitudes, the ancestral genetic environment, and the different lengths of time in which they have lived at high-altitudes must have given rise, by natural selection, to different genetic adaptations for Tibetan and Andean populations. Tibetan highlanders have acquired, evolutionarily, a positive selection of genes involved in the regulation of erythropoiesis. Thus, Tibetans have acquired the physiological ability to maintain hemoglobin concentrations at levels similar to those of sea-level inhabitants. In contrast, the Andean Aymaras did not appear to have evolved mechanisms to modify erythropoiesis but have adapted to exist with higher hemoglobin concentrations than individuals dwelling at sea level. This different phenotype is likely due to the positive selection of different genes, but it also may reflect the influence of a smaller number of generations and a shorter period of exposure to high altitudes by Aymaras [6,7].

Erythrocytosis is one of the more prevalent hematological disorders worldwide, with significant clinical and sociological relevance. The prevalence of high altitude erythrocytosis differs according to the region, the duration of residence and the altitude of residence, and specific evolutionary adaptations unique to a particular ethnic group. High-altitude erythrocytosis prevalence differs greatly among high mountain dwellers; while it is found in only 1.2% of Native Tibetans, it is reported to be present in 5.6% of Han Chinese and 15.6% of Aymara highlanders [6,7]. Hemoglobin levels in Aymaras vary and generally increase with increasing altitudes. In Santa Cruz (400 m) Hb levels range from 12 to 15 g/dL in women and 13 to 16 g/dL in men. In La Paz (4000 m), Hb levels range from 14 to 17 g/dL in women and 15 to 18 g/dL in men; and in Chorolque (5000 m), Hb levels range from 15 to 18 g/dL in women and 16 to 19 g/dL in men [6,7].

We and others, using genome-wide association studies (GWAS) and whole exome sequencing, have identified highly selected genetic haplotypes in two genes, *EPAS1* (encoding hypoxia-inducible factor 2-α [HIF-2α]) and *EGNL1* (encoding prolyl hydroxylase 2 [PHD2]) in Tibetans [18,19]). The HIF proteins, HIF-1 and HIF-2 are heterodimeric transcription factors that utilize different α-subunits and the same β-subunit to mediate the hypoxic response. The haplotype of the *EPAS1* gene entered the Tibetan genome from ancient Denisovan introgression and then underwent further non-Denisovan evolution [20]. PHD2 is the principal negative regulator of the stability of HIF-α -subunits. Two *EGLN1* variants, PHD2^D4E^ and PHD2^C127S^, occur in *cis* and form a linked haplotype in Tibetans that has increased activity in hypoxia and downregulates HIF-1 and HIF-2 levels [21]. The PHD2^D4E:C127S^ haplotype correlates with lower Hb in Tibetan highlanders, and this correlation is even more marked when the haplotype is coinherited with a C/C genotype of non-Denisovan *EPAS1* SNP rs142764723, which is an intronic variant [22].

There are enriched genes in Andean Aymaras (*BRINP3*, *NOS2*, *SH2B1*, and *TBX5*) and *PYGM,* which are involved in the regulation of cardiovascular development, but we have not found obvious correlations with Hb levels in Aymaras living at high altitudes [23,24]. However, this has not been studied in a larger sample or at different altitudes of residence. BRINP3 (rs11578671) is related to vascular inflammation that may be relevant in myocardial infarction risk. NOS2 (rs34913965) deals with the production of nitric oxide in a wide variety of tissues and regulates nitrosative stress. SH2B1 (rs12448902) encodes an adapter protein involved with several tyrosine kinases including JAK2, a negative regulator of the erythropoietin-signaling pathway. TBX5 (rs10744822) is involved in heart development, especially of the septum and conduction system. PYGM (rs487105) is involved in cardiac development and specification of limb identity [24].

We interrogated the evolutionary-selected gene regions that are shared between Aymaras and Tibetans and those solely enriched in Aymaras for their possible effects on numbers of blood cell [5]. We postulated that these gene variants might provide an advantage in highlanders by improving fitness at high altitudes [25,26,27].

## 2. Methods and Materials

### 2.1. Sample Acquisition

We studied two Tibetan-enriched haplotypes (*EGLN1* and *EPAS1*), as well as five Aymara-enriched haplotypes (*BRINP3*, *NOS2*, *SH2B1*, *TBX5*, and *PYGM*) in Bolivian Andean Aymaras residing at three different altitudes: 400 m (Santa Cruz), 4000 m (La Paz), and 5000 m (Chorolque), and compared them with previously published analyses of 347 Tibetans living at altitudes ranging from 200 to 4300 m [22]. After obtaining institutional review board approval from each collaborating group, we recruited 177 unrelated volunteers residing at La Paz (*n* = 124), Santa Cruz (*n* = 43), and Chorolque (*n* = 10). Samples from Europeans (*n* = 10) residing at 4000 m over 10 years were also interrogated.

Written informed consent was obtained from all participants. Demographic data, including age, gender, and ethnicity, were recorded. Peripheral blood was collected using acid citrate buffer (ACD) or ethylene diamine tetra acetic acid (EDTA) tubes. A complete blood count was performed.

Allele frequencies of European, Peruvian, and Columbian populations were obtained from the 1000 Genomes database [28].

### 2.2. DNA Isolation

Genomic DNA from granulocytes and mononuclear cells was isolated using the PUREGENE DNA Purification Kit (Qiagen, Valencia, CA, USA). In samples with limited genetic material, whole genome amplification (WGA) was performed (REPLI-g kit, Qiagen). DNA quantity and quality were determined by UV spectroscopy (ND-1000, NanoDrop Technologies, Wilmington, DE, USA) and adjusted to 50 ng/μL in 10 mM Tris pH 8.0, 0.1 mM EDTA (TE buffer).

### 2.3. EGLN1 Exon 1 Sanger-Based Genotyping

Exon 1 of *EGLN1* is GC rich, poorly detectable by whole-genome sequencing, and analysis is generally not possible by conventional Sanger sequencing [21]. To improve the Sanger sequencing method for *EGLN1* genotyping, we amplified the 5′ portion of *EGLN1* exon 1 (c.-118_c.471) using Platinum^®^ *Taq* DNA Polymerase (Life Technologies), FailSafe™ PCR 2X PreMix “J” (Epicentre, Madison, WI, USA), and M13-tailed primers: 5′-TGT AAA ACG ACG GCC AGC ATG GCG CAG TAA CG and 5′-CAG GAA ACA GCT ATG ACC TGG AAC AGC GAT GAG. The PCR conditions were: initial denaturing at 98 °C for 30 s, followed by 10 cycles of 20 s denaturing at 98 °C, 20 s annealing at 62 °C minus 0.5 °C/cycle and 1 min extension at 72 °C, and 25 cycles of 20 s denaturing at 98 °C, 20 s annealing at 57 °C and 1 min extension at 72 °C. Amplification products were purified using EXOSapIt (Affymetrix, Santa Clara, CA, USA) to remove excess PCR primers and dNTPs. Sequencing was performed using M13 primers and the BigDye Terminator v3.1 Cycle Sequencing Kit (Life Technologies, Grand Island, NY, USA) following the manufacturer’s published protocol using a 3130xl Genetic Analyzer (Life Technologies). Sequence data were analyzed using Mutation Surveyor software (Softgenetics, State College, PA, USA).

### 2.4. EPAS1/HIF2a SNP Genotyping

The ten selected Tibetan SNPs were amplified using multiplex, touch-down PCR and analyzed using the single nucleotide extension (SNE) protocol (Life Technologies). Multiplex PCR was run with Platinum^®^ *Taq* DNA Polymerase (Life Technologies), FailSafe™ PCR 2X PreMix “D” (Epicentre), and the primers listed in Supplementary Table S2. The PCR conditions were: an initial denaturation at 98 °C for 30 s followed by 10 cycles of step-down primer annealing using 20 s denaturing at 98 °C, 20 s annealing at 62 °C minus 0.5 °C/cycle, and 45 s extension at 72 °C, followed by 25 cycles of 20 s denaturing at 98 °C, 20 s annealing at 57 °C and 45 s extension at 72 °C. Amplification products were purified using EXOSapIt (Affymetrix) to remove excess PCR primers and dNTPs. Amplicons were then added to the SNaPshot Multiplex System (Life Technologies) reagents according to the manufacturer’s protocol. Following the SNE cycling reaction, excess ddNTPs were removed using shrimp alkaline phosphatase (Affymetrix, Santa Clara, CA, USA), and the products were analyzed on a 3130xl Genetic Analyzer (Life Technologies). Primers for PCR were designed using MPprimer [29]. Primers for SNE were manually selected and screened using Primer Designer (v.5) software (Scientific & Educational Software, Morrisville, NC, USA). The SNE primers were designed to have lengths between 15 and 60 nucleotides (Table S1). Fragment analysis was conducted using GeneMarker software (Softgenetics).

### 2.5. Genotyping Method for Five Aymara Selected SNPs [23]

rs34913965 (assay ID: C_2593699_10), rs12448902 (assay ID: C_372211_10), and rs487105 (assay ID: C_188913350_10) were genotyped by TaqMan genotyping assay (Thermofisher). rs11578671 and rs10744822 were genotyped using the restriction enzyme method. For rs11578671, 299 base pairs (bps) of the *BRINP3* gene were amplified using primers 5′ GGC GCA CAC CTG TAG TCC 3′ and 5′ CCC TCT GTG TTC AGT ATT ATT CAT TTT 3′ using HotStarTaq (Qiagen) with The PCR conditions were: an initial denaturation at 95 °C for 15 min, followed by 35 cycles of denaturing at 95 °C for 40 s, annealing at 61.7 °C for 40 s, and extension at 72 °C for 30 s, and then a second extension stage at 72 °C for 10 min. Restriction enzyme, BtsI (New England Biolabs), was added to the PCR product and incubated for 1 h and 30 min at 37 °C. The size of restricted PCR products was loaded in 1.5% agarose gel. G/G (299 bps), G/A (299, 99, and 200 bps), and A/A (99 and 200 bps) genotypes were identified by different sizes. For rs10744822, 294 bps of the *TBX5* gene were amplified by PCR using primers 5′ CAG AGG CAG GTG GAT CAC T 3′ and 5′ TCC TTG ACC AGC CTC ATT GT 5′. The same PCR condition for s10744822 genotyping was used. BstUI (New England Biolabs) was used to cut the “C” allele. The PCR product was incubated at 60 °C for 1 h and 30 min. Size of restricted PCR products was loaded in 1.5% agarose gel. C/C (114 and 180 bps), G/T (294, 114, and 180 bps), and T/T (294 bps) genotypes were identified by different sizes of products. An example of gel images is presented in Figure S1.

### 2.6. Statistical Analysis

*P*-values for allele frequencies were calculated by Fisher’s exact test. Hematocrit, hemoglobin, white blood cell counts, and platelet counts were presented as the mean ± standard deviation (SD). The *p*-value was calculated by unpaired *t*-test. Statistical analysis was performed using GraphPad Prism Software (version 9.5.1).

## 3. Results

### 3.1. Aymaras Share Two of Five Non-Denisovan EPAS1 SNPs That Are Selected in Tibetans

Five non-Denisovan-like *EPAS1* SNPs enriched in Tibetan highlanders displayed lower prevalence in Tibetan lowlanders [22]. Only two of these SNPs were present in Aymara highlanders. One SNP, the rs130005507 G allele, had a frequency of 0.35 in Aymara highlanders, a lower prevalence than Tibetan highlanders (gene frequency 0.755), which is comparable with two South American populations (Peruvian and Colombian) and Europeans (Table 1). Another SNP associated with decreased Hb concentration in Tibetans, the rs142764723 G allele, had a frequency of 0.04 in Aymara highlanders whereas the frequency was 0.480 in Tibetan highlanders. Tibetan Denisovan-like *EPAS1* variants were not present in Aymara highlanders.

### 3.2. The PHD2^C127S^ Haplotype in Heterozygous form Had Lower Prevalence in Aymaras

Tibetan-selected *EGLN1* haplotypes had distinct prevalence patterns between Aymaras and Tibetan highlanders. The Aymara highlanders did not carry the Tibetan-specific *EGLN1* PHD2^D4E^ mutation. The *EGLN1* PHD2^C127S^ SNP had a frequency of 0.11 in Aymara highlanders, which was not significantly different from two other South American populations (Peruvian and Colombian) and Europeans (Table 1). The PHD2^C127S^ SNP had a frequency of 0.870 in Tibetan highlanders and usually occurred in linkage with PHD2^D4E^. The prevalence of these haplotypes increased with increasing altitude of residence in Tibetans [22].

### 3.3. Cardiovascular Enriched SNPs in Aymaras

The *BRINP3* (rs11578671), *NOS2* (rs34913965), *SH2B1* (rs12448902), *TBX5* (rs10744822), and *PYGM* (rs487105) haplotypes were enriched in both Aymara lowlanders and Aymara highlanders; these variants were present at lower prevalence in Europeans (Table 1). Allele frequencies of *BRINP3* (rs11578671), *NOS2* (rs34913965), and *TBX5* (rs10744822) increased at higher elevations (Table 2). Female heterozygotes for *BRINP3* rs11578671 had higher Hb levels compared to wild type and homozygotes (Table 3). Individuals with *NOS2* (rs34913965) had higher leukocyte and platelet counts compared to those without it (Table 4 and Table 5). Leukocytes were higher in individuals with *SH2B1* rs12448902 (Table 4).

## 4. Discussion

High-altitude dwellers have adapted to chronic high-altitude hypoxia in several locations around the world, and a large number of studies support the idea that human adaptation at high altitudes has a genetic basis. In this way, *EPAS1* and *EGLN1* haplotypes are related to high altitude adaptation of Hb but only in Tibetans [18,19,21]. These adaptations occur in the hypoxia-inducible factor signaling pathway and blunt erythropoiesis. In the hypoxia-inducible factor pathway, HIF-α subunits are hydroxylated by PHD2 and marked for degradation by proteasomes under normoxia, whereas α subunits of HIF are stabilized in hypoxia. HIF-2 induces the transcription of several genes involved in erythropoiesis, including erythropoietin, leading to an increased Hb level [30,31]. However, a non-Denisovan like *EPAS1* SNP, rs142764723 CC, seems to facilitate HIF-α degradation by proteasomes even under hypoxia, especially when co-inherited with the *EGLN1* PHD2^D4E:C127S^ haplotype; therefore, Hb levels may be lower than expected for the degree of elevation [32]. Tibetan highlanders have a higher prevalence of non-Denisovan *EPAS1* SNPs than Tibetan lowlanders [22]. Aymara highlanders have only two of the five non-Denisovan SNPs present in Tibetans and no Denisovan-like *EPAS1* SNPs. Allele frequencies of two non-Denisovan EPAS1 SNPs in Aymaras were not different from those of two South American populations (Peruvian and Colombian) and Europeans. These data do not provide evidence of convergent evolution between Aymaras and Tibetans. We show that, unlike Tibetans, the Aymaras have not undergone significant evolutionary genetic selection in these HIF pathway components that would alter their elevated Hb levels at high altitude. Thus, other genomic determinants for Aymara’s high-altitude adaptation will need to be ascertained in the future.

The regulation of erythropoiesis and the amount of hemoglobin in red cells is regulated by the oxygen delivery to the tissues. Erythropoiesis is regulated by stimulation of erythropoietin by hypoxia-inducible factors (HIFs), which are crucial components of the tissue oxygen sensor [33,34]. While erythropoietin is the principal component of a physiological response to hypoxia, oxygen delivery to tissue is also regulated by the affinity of hemoglobin and oxygen.

There are mutations of alpha, beta, and or gamma, globin genes that result in hemoglobins with high-affinity for oxygen [35]. These high-affinity hemoglobins lead to decreased oxygen delivery to the tissue and compensatory erythrocytosis. In contrast there are also low-oxygen affinity hemoglobin mutations resulting in superior oxygen delivery to tissue. In these low-oxygen affinity hemoglobin mutants, the activity of the oxygen sensor and HIF levels are appropriately downregulated, resulting in decreased erythropoiesis, thereby maintaining normal functional oxygen delivery but decreasing Hb levels (laboratory anemia). This “*laboratory anemia*” is at times referred to as “*the absence of functional anemia*” [36,37]. In early studies of Tibetan adaptation, it was postulated that Tibetans may compensate for environmental hypoxia by selecting for hemoglobin with low oxygen affinity, but this hypothesis has been conclusively ruled out [27]. We and others also searched for the low-oxygen affinity hemoglobins in Aymaras and were unable to find them (unpublished data, Amaru et al.).

Oxygen delivery to the tissues could also be moderated by diphosphoglycerate (2,3 DPG) levels that modulate hemoglobin oxygen affinity. While the level of 2,3 DPG is influenced by pH, there are also genetic variants of diphosphoglycerate mutase (BPGM) that affect 2,3-DPG levels. Very few of these rare loss-of-function BPGM mutations that result in increased erythrocytosis have been described [38,39].

Further along the glycolytic pathway, downstream of 2,3 DPG synthesis, is the enzyme pyruvate kinase (PK). It exists in two forms, encoded by two genes located on different chromosomes. One of them is the *PKLR* gene that regulates pyruvate kinase enzyme activity in red cells [40]. Loss-of-function mutations of the *PKLR* gene lead to the most common form of chronic hemolytic anemia [41]. Decreased PK activity results in increased levels of 2,3 DPG with concomitant higher oxygen delivery to the tissues. Thus, individuals with PK deficiency have less severe symptoms than their hematologic indices suggest. Genome analysis of Tibetans revealed that *PKLR* is one of the evolutionarily selected genes in Tibetans [18]. We previously described that a *PKLR* variant present in most Tibetans is associated with a decreased transcript of *PKLR* in the erythroid progenitors, and presumably with decreased PK enzyme activity and a higher level of 2,3 DPG [27,42]. This variant is also present in Aymaras at a frequency of about 44% [42]. This variant may change the hemoglobin oxygen dissociation curve and improve oxygen delivery to the tissues. Future studies to measure Hb oxygen affinity are planned to elucidate the effect of this *PKLR* variant in Aymara people. Thus, the assessment of this selected *PKLR* haplotype on the hemoglobin dissociation curve in Aymaras will have to await further studies.

The bone morphogenetic protein/retinoic acid-inducible neural-specific 3 (*BRINP3*) gene regulates osteoblast differentiation [43]. Variants of this gene have been associated with myocardial infarction [44] and aggressive periodontitis [45]. BRINP3 also plays a role as a tumor suppressor gene [46,47]. Upregulation of the *BRINP3* gene is also involved in augmented inflammation via the NF-kB pathway and reactive oxygen species [48], and inflammation leads to suppression of erythropoiesis [49]. We show here that rs11578671, located upstream of *BRINP3,* is associated with higher Hb when present in heterozygous individuals. This variant may alter *BRINP3* expression, resulting in augmented erythropoiesis; however, its possible role in increasing erythropoiesis by diminishing inflammation remains speculative at this time.

*NOS2* encodes nitric oxide synthase (NOS). Inhibiting NO synthesis using an L-arginine analog increases leukocytes in rats [50]. Tibetans have higher NO levels in their lungs compared to Aymaras [51], who have similar levels as lowlanders at sea level. We show here that *NOS2* rs34913965 is associated with higher leukocyte counts in Aymaras. This variant might reduce NO synthesis by suppressing *NOS2* expression, resulting in an increased number of leukocytes. Individuals with this variant also have higher platelet counts. NO also inhibits platelet activation, adhesion, and aggregation [52]. NO treatment induces apoptosis of megakaryocytes but also plays a role in terminal megakaryocytopoiesis (platelet release phase) [53]. This variant might alter NO production, resulting in increased megakaryocytopoiesis.

*SH2B1* (Src-homology 2B adaptor protein 1) encodes an adaptor protein with an SH2-domain and inhibits erythropoietin-signaling by binding to the erythropoietin receptor [54]. In our analysis, *SH2B1* rs12448902 is associated with higher leukocytes in Aymaras. Unfortunately, we were unable to get data on differential leukocyte components, such as B and T lymphocytes, monocytes, and granulocytes, precluding more detailed analyses of the effect of these *NOS2* and *SH2B1* haplotypes on different leukocyte compartments. More specific leukocyte component associations will need to await future studies.

In conclusion, the high-altitude adaptation of the Andean Aymara people remains an open line of investigation. Although certain Tibetan-enriched haplotypes of *EPAS1* and *EGLN1* are present in Aymaras, these SNPs appear to have contributed only slightly to the same high-altitude adaptation mechanism as observed in Tibetans. This suggests that the Tibetans and Aymaras have undergone non-convergent genomic adaptation accounting for their successful existence in extremely hypoxic high-altitude environments. Tibetan adaptation is closely related to hematological issues, whereas Aymara adaptation is related to cardiovascular effects. Moreover, it is likely that these haplotypes might be present in other high-altitude dwellers, likely related to the length and duration of residence at high altitude. To determine the genotype association with tissue-specific physiological measurements, genetically modified cell lines and animal models will be required for future studies. More work is needed to ascertain the genetic determinants of Aymaran high-altitude adaptation.

## Figures and Tables

**Table 1:** Allele frequencies in Aymara, Peruvian, Colombian, and European populations.

	Gene SNP ID	Allele	Aymara	Peruvian	Colombian	European	Aymara vs. Peruvian	Aymara vs. Colombian	Aymara vs. European
Aymara selected SNPs	*BRINP3* rs11578671	G	0.20	0.43	0.83	1.00	0.0007 *	<0.0001 *	<0.0001 *
		A	0.80	0.57	0.17	0.00			
	*NOS2* rs34913965	C	0.17	0.39	0.71	0.90	0.0009 *	<0.0001 *	<0.0001 *
		T	0.83	0.61	0.29	0.10			
	*SH2B1* rs12448902	C	0.10	0.29	0.59	0.50	0.0011 *	<0.0001 *	<0.0001 *
		G	0.90	0.71	0.41	0.50			
	*TBX5* rs10744822	C	0.88	0.59	0.26	0.15	<0.0001 *	<0.0001 *	<0.0001 *
		T	0.12	0.41	0.74	0.85			
	*PYGM* rs487105	G	0.34	0.42	0.79	0.80	0.3078	<0.0001 *	<0.0001 *
		A	0.66	0.58	0.21	0.20			
Tibetan selected SNPs	*EGLN* rs186996510	C	1.00	-	-	1.00			1.0000
		G	0.00	-	-	0.00			
	*EGLN1* rs12097901	G	0.89	0.87	0.86	0.92	0.8282	0.6696	0.6306
		C	0.11	0.14	0.14	0.08			
	*EPAS1* rs13005507	G	0.35	0.46	0.34	0.39	0.1495	1.0000	0.6605
		C	0.65	0.54	0.66	0.61			
	*EPAS1* rs142764723	A	0.96	0.98	1.00	0.99	0.6827	0.1212	0.3687
		G	0.04	0.02	0.00	0.01			

*p*-value was calculated by Fisher’s exact test; * *p*-value < 0.05; 1000 genome data was used for allele frequencies of *EPAS1* SNPs of Europeans.

**Table 2:** Allele frequencies in Aymaras at different altitudes.

	Gene SNP ID	Allele	Aymaras			*p*-Values	*p*-Values	*p*-Values
			400 m	4000 m	5000 m	400 m vs. 4000 m	400 m vs. 5000 m	4000 m vs. 5000 m
Aymara selected SNPs	*BRINP3* rs11578671	G	0.20	0.29	0.10	0.1881	0.0734	0.0011 *
		A	0.80	0.71	0.90			
	*NOS2* rs34913965	C	0.23	0.19	0.10	0.6029	0.0212 *	0.1070
		T	0.77	0.81	0.90			
	*SH2B1* rs12448902	C	0.08	0.12	0.10	0.4804	0.8056	0.8217
		G	0.92	0.88	0.90			
	*TBX5* rs10744822	C	0.86	0.78	1.00	0.1972	<0.0001 *	<0.0001 *
		T	0.14	0.22	0.00			
	*PYGM* rs487105	G	0.40	0.27	0.35	0.0718	0.5592	0.2845
		A	0.60	0.73	0.65			
Tibetan selected SNPs	*EGLN* rs186996510	C	1.00	1.00	1.00	1.0000	1.0000	1.0000
		G	0.00	0.00	0.00			
	*EGLN1* rs12097901	G	0.90	0.90	0.88	1.0000	0.8217	0.8217
		C	0.10	0.10	0.12			
	*EPAS1* rs13005507	G	0.39	0.22	0.45	0.0137 *	0.4739	0.0009 *
		C	0.61	0.78	0.55			
	*EPAS1* rs142764723	A	1.00	0.93	0.95	0.0140 *	0.0594	0.7673
		G	0.00	0.07	0.05			

*p*-value was calculated by Fisher’s exact test; * *p* value < 0.05.

**Table 3:** Correlation of genotypes to hemoglobin (Hb) and hematocrit (Hct) at 4000 m.

	Gene SNP ID	Gender	Hb (g/dL)			*p* Values		
			WT	HET	HOMO	WT vs. HET	WT vs. HOMO	HET vs. HOMO
Aymara selected SNPs	*BRINP3* rs11578671	F	13.9 ± 0.7	17.0 ± 4.9	14.2 ± 3.8	0.0187 *	0.660	0.057
		M	16.9 ± 2.1	18.3 ± 3.4	18.1 ± 2.9	0.263	0.258	0.768
	*NOS2* rs34913965	F	11.8	15.9 ± 4.5	15.2 ± 4.3	-	-	0.658
		M	18.1 ± 1.6	18.3 ± 3.3	17.9 ± 2.9	0.862	0.881	0.534
	*SH2B1* rs12448902	F	12.8	14.1 ± 4.1	15.2 ± 3.8	-	-	0.444
		M	19.8 ± 1.9	18.4 ± 3.4	17.9 ± 3.0	0.508	0.286	0.654
	*TBX5* rs10744822	F	NONE	15.8 ± 4.0	15.0 ± 4.6	-	-	0.599
		M	18.2 ± 2.9	17.9 ± 2.8	18.0 ± 3.2	0.784	0.849	0.915
	*PYGM* rs487105	F	16.3 ± 4.5	13.3 ± 3.6	14.5 ± 3.9	0.553	0.415	0.066
		M	17.8 ± 2.5	18.9 ± 4.1	17.6 ± 2.6	0.363	0.821	0.270
Tibetan selected SNPs	*EGLN1* rs12097901	F	15.8 ± 2.1	16.0 ± 0.8	NONE	0.901	-	-
		M	17.8 ± 1.9	19.3 ± 3.1	NONE	0.135	-	-
	*EPAS1* rs13005507	F	16.6 ± 0.6	16.6	18.4	-	-	-
		M	18.8 ± 2.1	18.2 ± 1.4	18.7	0.610	-	-
	*EPAS1* rs142764723	F	16.7 ± 0.8	17.6	NONE	-	-	-
		M	18.7 ± 1.6	17.8 ± 2.5	NONE	0.519	-	-
	**Gene** **SNP ID**	**Gender**	**Hct (%)**			***p*-Values**		
			**WT**	**HET**	**HOMO**	**WT vs. HET**	**WT vs. HOMO**	**HET vs. HOMO**
Aymara selected SNPs	*BRINP3* rs11578671	F	45.4 ± 3.5	52.9 ± 15.5	45.0 ± 10.4	0.077	0.950	0.063
		M	55.3 ± 6.7	58.0 ± 10.3	56.1 ± 9.8	0.488	0.822	0.438
	*NOS2* rs34913965	F	39.2	50.7 ± 14.4	47.4 ± 12.5	-	-	0.521
		M	59.3 ± 5.2	58.3 ± 10.1	55.4 ± 9.7	0.838	0.390	0.237
	*SH2B1* rs12448902	F	42.1	45.1 ± 11.8	47.6 ± 11.2	-	-	0.549
		M	63.1 ± 6.2	57.0 ± 11.1	56.3 ± 9.6	0.379	0.231	0.837
	*TBX5* rs10744822	F	12.8	49.4 ± 12.5	47.6 ± 13.2	-	-	0.660
		M	55.4 ± 10.4	57.0 ± 9.2	56.7 ± 9.8	0.627	0.685	0.887
	*PYGM* rs487105	F	50.6 ± 13.9	43.9 ± 11.1	45.5 ± 10.7	0.787	0.443	0.175
		M	55.2 ± 8.4	59.8 ± 12.8	56.2 ± 7.6	0.422	0.727	0.154
Tibetan selected SNPs	*EGLN1* rs12097901	F	46.8 ± 3.8	46.6 ± 4.9	NONE	0.946	-	-
		M	54.6 ± 5.8	60.3 ± 9.9	NONE	0.068	-	-
	*EPAS1* rs13005507	F	47.7 ± 2.6	48.2	52.7	-	-	-
		M	54.0 ± 6.6	52.1 ± 4.3	53.7	0.595	-	-
	*EPAS1* rs142764723	F	47.9 ± 2.7	51.4	NONE	-	-	-
		M	53.5 ± 4.9	51.7 ± 9.1	NONE	0.676	-	-

Data are presented as mean ± standard deviation (SD); *p*-value was calculated by unpaired *t*-test; * *p*-value < 0.05. F: Females, M: Males. *p*-values could not be determined due to no or only one individual with WT, HET, or HOMO. NONE: No individual with this genotype.

**Table 4:** Correlation of genotypes to whole blood cell counts.

	Gene SNP ID	Leukocyte Counts (/μL)			*p*-Values		
		WT	HET	HOMO	WT vs. HET	WT vs. HOMO	HET vs. HOMO
Aymara selected SNPs	*BRINP3* rs11578671	6716.7 ± 2577.1	5737.0 ± 1451.6	5675 ± 1500.8	0.228	0.199	0.829
	*NOS2* rs34913965	5066.7 ± 527.9	6005.6 ± 1768.0	5761.0 ± 1613.5	0.0158 *	0.0266 *	0.463
	*SH2B1* rs12448902	5075.0 ± 330.4	5738.1 ± 1605.1	5802.1 ± 1654.3	0.101	0.0095 *	0.872
	*TBX5* rs10744822	5576.9 ± 1464.7	5746.3 ± 1330.2	5870.0 ± 1821.4	0.698	0.586	0.682
	*PYGM* rs487105	5819.1 ± 1760.5	5743.3 ± 1768.8	5593.3 ± 1151.1	0.768	0.637	0.845
Tibetan selcted SNPs	*EGLN1* rs12097901	6314.3 ± 1587.1	5900.0 ± 1014.9	NONE	0.463	-	-
	*EPAS1* rs13005507	6840.0 ± 1190.9	6366.7 ± 2335.5	7100.0 ± 1979.9	0.653	0.788	0.707
	*EPAS1* rs142764723	6745.0 ± 1593.9	6700.0 ± 1417.7	NONE	0.964	-	-

Data are presented as mean ± standard deviation (SD); *p*-value was calculated by unpaired *t*-test; * *p*-value < 0.05. *p*-values could not be determined due to no or only one individual with WT, HET, or HOMO. NONE: no individual with this genotype.

**Table 5:** Correlation of genotypes to platelet counts.

	Gene	Platelet Counts (k/ μL)			*p*-Values		
		WT	HET	HOMO	WT vs. HET	WT vs. HOMO	HET vs. HOMO
Aymara selected SNPs	*BRINP3* rs11578671	305.7 ± 116.8	304.3 ± 226.3	303.0 ± 205.5	0.978	0.950	0.975
	*NOS2* rs34913965	195.2 ± 60.5	282.5 ± 284.4	319.3 ± 187.4	0.079	0.0018 *	0.431
	*SH2B1* rs12448902	287.0 ± 132.2	254.4 ± 116.6	300.1 ± 191.2	0.619	0.893	0.161
	*TBX5* rs10744822	296.9 ± 198.2	263.8 ± 110.3	326.4 ± 242.9	0.574	0.682	0.067
	*PYGM* rs487105	318.2 ± 232.7	250.7 ± 129.6	359.3 ± 226.0	0.101	0.536	0.070
Tibetan selcted SNPs	*EGLN1* rs12097901	300.8 ± 101.6	234.2 ± 97.5	NONE	0.085	-	-
	*EPAS1* rs13005507	344.3 ± 88.0	334.3 ± 90.2	371.0 ± 33.9	0.819	0.684	0.610
	*EPAS1* rs142764723	345.5 ± 83.5	334.3 ± 98.6	NONE	0.835	-	-

Data are presented as mean ± standard deviation (SD); *p*-value was calculated by unpaired *t*-test; * *p*-value < 0.05. *p*-values could not be determined due to no or only one individual with WT, HET, or HOMO. NONE: no individual with this genotype.

## Data Availability

For original data, please contact josef.prchal@hsc.utah or amaru.ricardo@icloud.com.

## Supplementary Materials

The following supporting information can be downloaded at: https://www.mdpi.com/article/10.3390/genes14030640/s1, Table S1: Amplification and SNE primers *EPAS1*/HIF-2α region of Chromosome 2; Figure S1: Example of gel image for BRINP3 (rs11578671) and TBX5 (rs10744822) genotyping assay using restriction enzymes.
