# Supplementary material for: “What We Know and What We Do Not Know about Evolutionary Genetic Adaptation to High Altitude Hypoxia in Andean Aymaras”
Source: Genes (Basel). 2023 Mar 3;14(3):640. doi: 10.3390/genes14030640 (PMC10048644; doi:10.3390/genes14030640)
Supplement: Supplementary file 1 [file genes-14-00640-s001.zip › genes-2228531-Supplementary Figure S1.pdf]

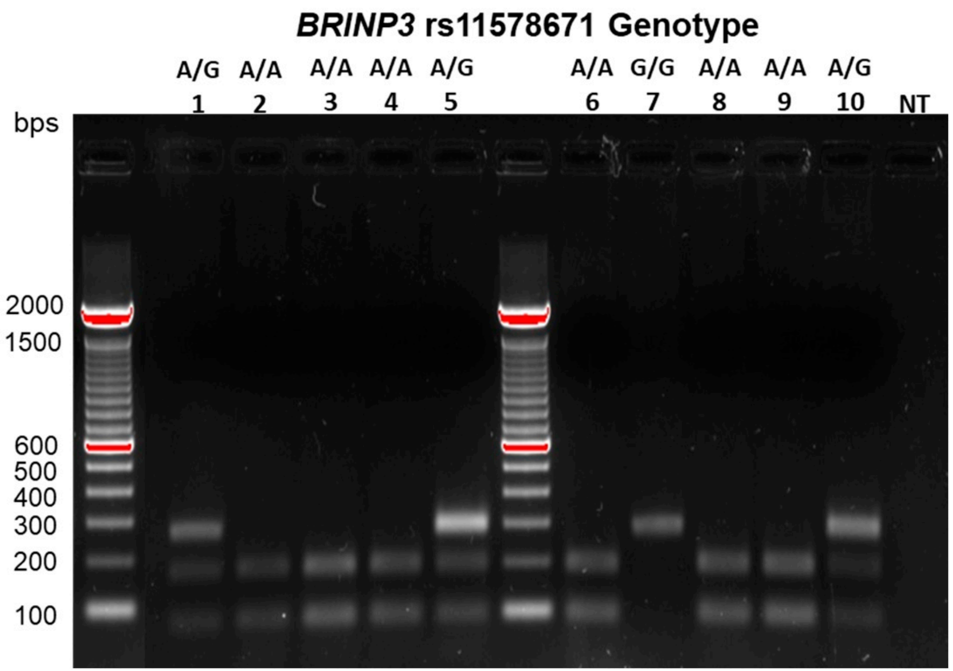


| Genotype | Expected size |
|---|---|
| A/A | 99/200 |
| A/G | 299/99/200 |
| G/G | 299 |

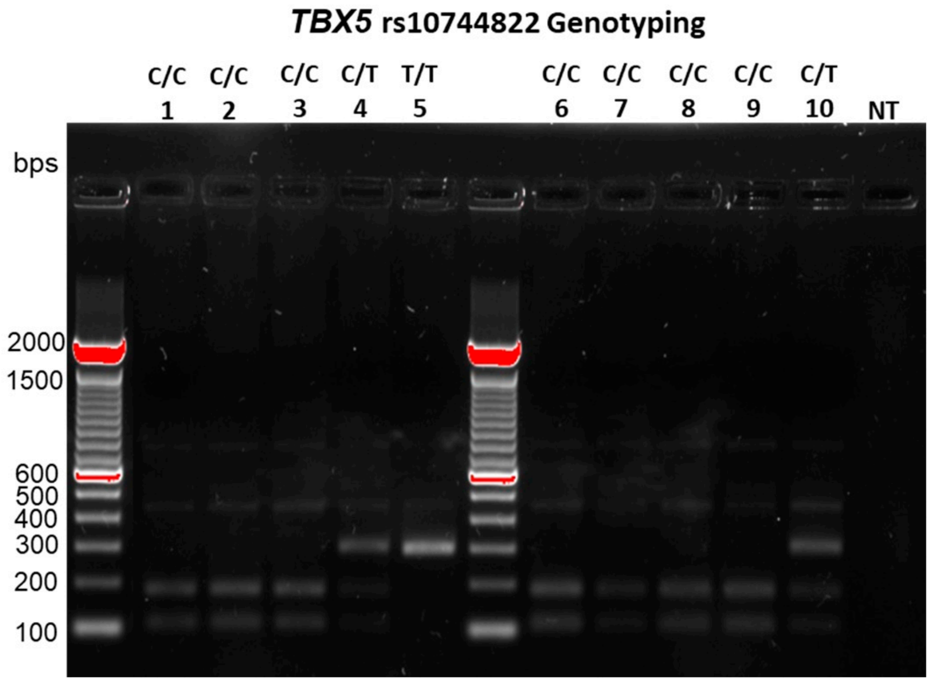


| Genotype | Expected size |
|---|---|
| C/C | 114/180 |
| C/T | 294/180/114 |
| T/T | 294 |

**Supplementary figure S1. Example of gel image for BRINP3 (rs11578671) and TBX5 (rs10744822) genotyping assay using restriction enzymes.**

Details of method are described in Methods and materials in the manuscript. NT: no template control
