# Supplementary material for: “What We Know and What We Do Not Know about Evolutionary Genetic Adaptation to High Altitude Hypoxia in Andean Aymaras”
Source: Genes (Basel). 2023 Mar 3;14(3):640. doi: 10.3390/genes14030640 (PMC10048644; doi:10.3390/genes14030640)
Supplement: Supplementary file 1 [file genes-14-00640-s001.zip › genes-2228531-Supplementary Table S1.pdf]

Supplementary Table S1: Amplification and SNE primers *EPAS1*/HIF-2α region of Chromosome 2.

Target	Forward Primer	Reverse primer	SNE primer
rs113305133	TATGCAAGGTT AGTG	CATGCTATTCCAA GG TTTCCCC	T(7)GCCCCTTACCTTCACCCC
rs149306391	GAGTATTCGCC TAGG	TCACTGTTCCCCTT TC ACAGAT	AGATTGTTATTGTCATTGTTCTTCT TT
rs188801636	AAAGGCCTTAT CCTCT AGCCTG	ACATCATTTCTCCA GA AGCCTA	TACATCATTTCTCCAGAAGCCTATA TT TAATATAA
rs61151542	TCCCGTTAATT ACAG	AACTCAGATTTCA GT GCCTTCG	T(14)CCTTGTGTTTTCCTCAAATACT A CAT
rs77111769	TTTGACCTTCC CCATT	CAGTTCTGGGCTG TA TTATGCC	T(23)CCCTTCTTAGGCAAACTAATGG
rs373417600	AGGATGAAAA CGCTC	ACTCTGTCTCTCCT GT TAAGCC	ATAAGGTTTAGTTTGTTCCCCACTT AC GTAACTGCTTTTAATAATGTGCCTT
rs150877473	GAGTCCCAGGT GTAG GGTAAC	GGGCAGTTGTTGTA G ACTTTCA	CGGCTCCATGTCTGACCCTTCCAC GC CTGT
rs142764723	AGAAAGCTTGT ACAG	GTTTTTCCTGTGCT GT TCTTGC	GAATCATGGGCTTGGGTTTTTCCT GT TTTGTTCTTGCGCTAGTGAATAAGT
rs117813469	AACCTGGACTC AGGA	CACATCTCTTCCTT CC ATCGC	TGTCCTGGCCCTCGCT
rs13005507	GTTGGGCCCA GAGTT GATCT	AGATGGAGGGGAT G CTTTGATT	TGTTGATCTGCTGACCCACTT
